# Methylenetetrahydrofolate Reductase C677T Gene Polymorphism as a Risk Factor for Hypertension in a Rural Population

**DOI:** 10.1155/2020/4267246

**Published:** 2020-02-13

**Authors:** Rony Mario Candrasatria, Suko Adiarto, Renan Sukmawan

**Affiliations:** Department of Cardiology and Vascular Medicine, Faculty of Medicine, Universitas Indonesia-National Cardiovascular Center Harapan Kita, Letjen S. Parman Road Kav 87, Jakarta 11420, Indonesia

## Abstract

Hypertension remains a public health burden despite advances in its management. Hence, the search for further risk stratification tools and prevention and new treatment approaches continues. Methylenetetrahydrofolate reductase (MTHFR) C677T polymorphism is associated with hypertension. Interestingly, riboflavin, as a cofactor of *MTHFR*, may control blood pressure in patients with mutant *MTHFR* variants. These double benefits of a risk stratification tool and treatment approach make it interesting. Because this polymorphism depends on ethnicity and geographic region, we aimed to determine the association between *MTHFR* C677T gene polymorphism and hypertension in a rural Indonesian-Sundanese population. This population-based case-control study included 213 hypertensive subjects and 202 nonhypertensive subjects as controls. The TaqMan assay was used to determine the *MTHFR* C677T genotypes. The odds ratio (OR) with 95% confidence interval (CI) was used to assess the risk of association. There was a significant difference in *MTHFR* C677T allele frequencies between the hypertensive and control groups (62.9% CC, 34.3% CT, 2.8% TT vs. 77.7% CC, 20.8% CT, 1.5% TT; *p*=0.004) and between mutant (TT and CT) and wild-type genotypes (CC) (*p*=0.001). The mutant genotype was associated with a risk of hypertension (OR 2.1; 95% CI 1.3–3.5) when adjusted for age, body mass index, waist circumference, and diabetes mellitus. The mutant of the *MTHFR* C677T gene increases the risk of hypertension in rural Indonesian-Sundanese population. These findings may be used in future studies to evaluate the effect of riboflavin supplementation in this population.

## 1. Introduction

Hypertension is a multifactorial disease influenced by interaction of genetic, environmental, and demographic factors [1]. Latest evidence showed that approximately 30%–50% of blood pressure variation was contributed by genetic factors [2]. Methylenetetrahydrofolate reductase (MTHFR) is an enzyme required to catalyze the conversion of 5,10-methylenetetrahydrofolate to 5-methyltetrahydrofolate, which is an important process in folate metabolism and remethylation of homocysteine to become methionine. Single-nucleotide polymorphism of the *MTHFR* C677T gene from alanine to valine reduces enzymatic activity, which, in turn, decreases the concentration of 5-methyltetrahydrofolate, which regulates the endothelial cells and nitrite oxides (NO) involved in the pathophysiology of hypertension [3, 4]. Several meta-analyses showed that *MTHFR* C677T polymorphism increased the risk of hypertension up to 24%–87% in whites and East Asians [5–9].

The reduced activity of MTHFR enzyme is related to the reduced concentration of its cofactor, riboflavin or vitamin B2. Supplementation of this widely available and affordable vitamin stabilizes the enzyme by increasing the endothelial functions and NO availability, especially in genotype variants [10]. Several randomized control trials showed significant blood pressure control in this variant population even after several years of follow-up [11–13].

This polymorphism depends on ethnicity and geographic region. No data showing an association of *MTHFR* C677T polymorphism with hypertension in Indonesians are available. The objective of this study was to determine the association of *MTHFR* C677T gene polymorphism with hypertension in rural Indonesian-Sundanese population.

## 2. Materials and Methods

This population-based case-control study had received approval from the ethics committee of National Cardiovascular Center Harapan Kita (approval number LB.02.01/VII/175/KEP.001/2017). This study was conducted from January to September 2017 in Gunung Sari village, West Java, Indonesia. Gunung Sari village is a rural area, approximately 90 km from the capital city Jakarta. The sample size was calculated from unmatched case-control formula. Sampling was performed with a simple stratified sampling method. All participants provided written informed consent.

The inclusion criteria for cases are adults aged ≥40 years, with hypertension (defined as repeated blood pressure ≥140 mmHg for systole and/or ≥90 mmHg for diastole, without regular medication), and with at least one of the traditional cardiovascular risk factors (e.g., smoking, obesity, and diabetes mellitus). Those with a history of symptomatic cardiovascular disease, cerebrovascular disease, and/or other systemic disease were excluded. Nonhypertensive subjects, defined as having repeated blood pressure ≤120 mmHg for systole and ≤80 mmHg for diastole and no history of hypertension, were included as controls.

Forty-seven health officers screened every house in Gunung Sari village using a standardized questionnaire, blood pressure examination, waist circumference measurement, and body mass index (BMI) assessment. Blood pressure was measured with a calibrated digital Omron HEM-711 (Kyoto, Japan) using the JNC7 protocol with at least two separate measurement occasions. Body weight was measured with a calibrated Camry weighing scale using the standardized protocol. Height was measured with standardized microtoise. Waist circumference was measured with a measuring tape measure using the World Health Organization protocol.

Selected subjects then underwent further blood examination. Twelve milliliters of ethylenediaminetetraacetic acid-based blood was obtained and underwent genotype assessment using the TaqMan assay. Genotyping was performed using Applied Biosystems® 7500 Real-Time PCR Systems (California, USA) with rs ID 1801133 and processed according to the manufacturer's instructions. The genotyping assay was performed in the molecular laboratory and stem cell facility of the National Cardiovascular Center Harapan Kita.

Data analysis was performed using SPSS version 23 (IBM Corp., Armonk, NY, USA). Categorical data are presented as number (*n*) and percentage (%). Continuous data are presented as mean ± standard deviation or median [range] if the data are nonnormally distributed. The chi-square test or Fisher's exact test was used for categorical data, and the unpaired *t*-test or Mann–Whitney *U* test was used for continuous data. The odds ratio (OR) and 95% confidence interval (CI) were calculated to estimate the association between various *MTHFR* genotypes and hypertension risk factors using the logistic regression test. A *p* value <0.05 was considered statistically significant. The chi-square test and Fisher's exact test were performed to test the Hardy–Weinberg equilibrium among each population surveyed, and the differences among the populations were compared with respect to allele and genotype frequencies.

## 3. Results

### 3.1. Characteristics of the Study Population

A total of 415 subjects, with 213 cases and 202 controls, were included. Cases tended to be older than the controls (median age: 52 vs. 45 years). Gender was distributed equally in both groups, with female predominance.

Cases had a higher rate of obesity than controls. Central obesity was also higher in cases than controls. No significant difference in smoking status between groups was observed. Cases had a higher number of diabetes mellitus than controls, but the difference was not statistically significant (Table 1).

### 3.2. Genotype Distribution of *MTHFR* C677T Polymorphism

**Figure 1:**
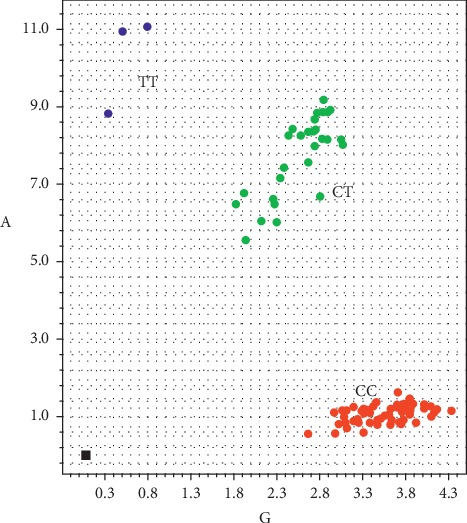
Allele discrimination plot. The genotype distribution of CC, CT, and TT was established according to the allele amplification number.

**Figure 2:**
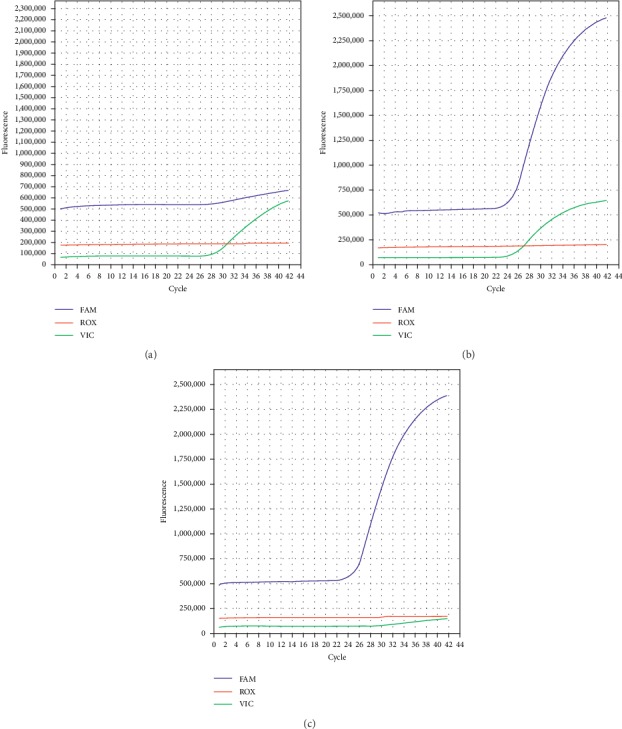
Fluorescence curve of the CC, CT, and TT genotype, from left to right, respectively.

The Hardy–Weinberg equilibrium test was performed to analyze the genotype distribution. In cases, the chi-square test was performed with *p* > 0.05. In controls, Fisher's exact test was performed with *p*=0.87. Hence, further analysis could be performed because the genotypes were in concordance with the Hardy–Weinberg equilibrium. Examples on how to assess the genotype are shown in Figures 1 and 2.

The genotype and allele frequency between groups was significantly different, with more mutant frequency observed in cases than controls (Table 2).

After conducting bivariate analysis, age, BMI, waist circumference, diabetes mellitus, and *MTHFR* polymorphism were included in the multivariate analysis using the double logistic regression test. In the first stage, diabetes was excluded because its *p* value was 0.7, with 95% CI of 0.4–3.3. The final model of multivariate analysis is presented in Table 3. Four predictors were found in this study: polymorphism of *MTHFR* C677T, age, BMI, and waist circumference. Polymorphism of *MTHFR* C677T significantly increased the risk of hypertension up to 2.1 times; BMI increased the risk of hypertension by 4.7 times; and waist circumference was related to hypertension. Meanwhile, age was found to be protective.

## 4. Discussion

To the best of our knowledge, this is the first study to investigate the association between *MTHFR* C677T polymorphism with hypertension in Indonesia, especially in a rural Sundanese population. The major finding of this study was that the presence of *MTHFR* variants was an independent predictor of hypertension.

Hypertension is influenced by genetic and environmental factors. The *MTHFR* C677T polymorphism is an interesting subject because of its advantage as a risk stratification tool and its potential for therapeutic intervention [4–9, 14, 15].

This case-control study was performed as a population-based study, which offers many advantages because of the homogeneity of the geographic region, ethnicity, and lifestyle similarities [16]. These may reduce biases when conducting an association study to assess risk factors.

From this study, we found that the CC genotype had the highest proportion in cases (62.9%), followed by CT (34.3%) and TT (2.8%). Although the mutant genotype frequency was high (37.1%) in this study, the homozygote mutant TT showed only 2.8%. This low proportion could be caused by recurrent early pregnancy loss (REPL), which prevented the survival of fetuses with the TT genotype. Several studies linked this gene polymorphism with REPL incidence up to 2–3 times through hyperhomocysteinemia and the absence of vitamin B-based supplementation [17–20]. There was a wide variation in the genotype frequency among different countries and even in the same country but with different ethnicities and geographic regions [21, 22].

After adjustment for age, BMI, waist circumference, and diabetes mellitus, we found that *MTHFR* C677T was associated with increased risk of hypertension (OR 2.1, 95% CI 1.3–3.5). This finding was similar to that of the study conducted by Wu et al. [7], which determined a significant association (allele TT + CT vs. CC: OR 1.34, 95% CI 1.24–1.46). Compared with the OR of previous studies, the OR of this study was higher, which was possibly because of the different geographic regions. The epigenetic factor could also influence this finding. Other predictors found significantly in this study were the traditional risk factors.

The genotyping method used in this study was the TaqMan assay, which was reported to have better sensitivity and accuracy than those of restriction fragment length polymorphism. This study was also in concordance with the Hardy-Weinberg equilibrium, which indicated that both groups had a homogenous population, less tendency to have genotyping error, proper random sampling, and adequate sample size [16].

In this study, diabetes mellitus status was established based on the memory of the subject of a history of confirmed diagnosis or treatment. Hence, the lack of laboratory confirmation was the limitation of this study.

In a study conducted by Horigan et al. [11], riboflavin supplementation reduced blood pressure significantly in hypertensive homozygote mutant variant. This study was conducted in Ireland with the white population. Hypothetically, a smaller effect will be observed in the heterozygote variant. In this study, the CT frequency reached 34.3%, a number high enough to stimulate a new study to seek association between riboflavin and hypertension. A randomized control trial to evaluate the effect of riboflavin supplementation on hypertension may be conducted in the future. Further studies in other regions of Indonesia are suggested.

## 5. Conclusion

Polymorphism of *MTHFR* C677T is associated with an increased risk of hypertension. This study shows the need for risk stratification and investigations into the potential preventive and therapeutic effect of riboflavin, either for personalized medicine or a community approach.

## Figures and Tables

**Table 1:** Clinical characteristics among cases and controls.

Characteristic	Cases (*n* = 213)	Controls (*n* = 202)	*p* value
Age (years)	52 [40–83]	45 [40–81]	<0.0001
Gender			
Male	50 (23.5)	51 (25.2)	0.7
Female	163 (76.5)	151 (74.8)	0.7
Blood pressure (mmHg)			
Systolic	174 [119–219]	115 [88–120]	<0.0001
Diastolic	103 [74–132]	73 [45–80]	<0.0001
Body mass index ≥25 kg/m^2^ (%)	146 (68.5)	68 (33.7)	<0.0001
Waist circumference (male, >90 cm; female, >80 cm)	157 (73.7)	89 (44.1)	<0.0001
Smoking	44 (20.7)	39 (19.3)	0.7
Diabetes mellitus	16 (7.5)	9 (4.5)	0.19

Values are presented as median [range] or *n* (%).

**Table 2:** Genotype and allele frequency of *MTHFR* C677T.

Group	Genotype (%)			Allele (%)	
	CC	CT	TT	Mutant (TT + CT)	Normal (CC)
Cases	134 (62.9)	73 (34.3)	6 (2.8)	79 (37.1)	134 (62.9)
Controls	157 (77.7)	42 (20.8)	3 (1.5)	45 (22.3)	157 (77.7)
*p* Value		0.004		0.001	
OR (95% CI)				2.1 (1.3–3.2)	

Values are presented as *n* (%).

**Table 3:** Final model of multivariate analysis.

Variable	B coefficient	*p* value	OR	95% CI
*MTHFR* C677T polymorphism (mutant vs. normal)	0.7	0.005	2.1	1.3–3.5
Age	−0.1	<0.0001	0.9	0.8–0.9
Body mass index	1.5	<0.0001	4.7	2.6–8.3
Waist circumference	0.7	0.017	1.9	1.1–3.4
Constant	4.152			

## Data Availability

The data used to support the findings of this study are available from the corresponding author upon reasonable request.
